# Comorbidities and Inflammation: How Chronic Diseases Prime the Host Response in Sepsis

**DOI:** 10.3390/ijms27167395

**Published:** 2026-08-19

**Authors:** Maria Vitória Oliveira Miguel, Rayssa Menon Santos, Matheus Marques de Oliveira, Gislaine Garcia Pelosi, Andressa Freitas

**Affiliations:** Department of Physiological Sciences, Center of Biological Sciences, State University of Londrina, Londrina 86055-970, Paraná, Brazilgislaine@uel.br (G.G.P.)

**Keywords:** sepsis, hypertension, metabolic syndrome, periodontitis, alcohol use disorder, psychosocial stress, inflammatory signaling, endothelial dysfunction, mitochondrial injury, multiple organ dysfunction syndrome

## Abstract

Considered a global public health priority, sepsis is characterized by life-threatening organ dysfunction caused by a dysregulated host response to infection. Its heterogeneous clinical presentation arises, in part, from pre-existing chronic conditions such as hypertension, metabolic syndrome, diabetes, alcohol exposure, psychosocial stress, and periodontitis, which induce persistent systemic changes even before the infectious event. This narrative review synthesizes evidence from experimental models and clinical studies to clarify the molecular and immunological mechanisms by which chronic conditions influence the septic state. We discuss how these conditions converge on common pathophysiological mechanisms, including low-grade chronic inflammation, oxidative stress, endothelial and mitochondrial dysfunction, and changes in the microbiota and neuroimmune regulation. Pathways such as TLR-NF-κB signaling and the NLRP3 inflammasome are maintained in a basal state of activation, lowering the threshold for hyperinflammatory responses and increasing the risk of multiple organ dysfunction syndrome. In conclusion, understanding these phenotypes can guide the identification of biomarkers and the development of personalized therapeutic strategies, thereby moving beyond one-size-fits-all approaches to the management of sepsis and septic shock.

## 1. Introduction

Sepsis remains a major challenge to global health, accounting for approximately 20% of all deaths worldwide [1,2]. According to the Sepsis-3 consensus, sepsis is defined as a dysregulated host response to infection resulting in life-threatening organ dysfunction [3]. Despite advances in early recognition and supportive care, survival rates have shown only modest improvement [4]. This persistent mortality highlights critical knowledge gaps in our understanding of how preexisting comorbidities influence host immune and molecular signaling and ultimately determine patient outcomes.

Chronic conditions such as hypertension, metabolic dysfunction (encompassing metabolic syndrome [MetS] and diabetes), psychosocial stress disorders, alcohol exposure, and periodontal disease trigger sustained alterations in immune regulation and vascular homeostasis, which precede and modulate the host response to infectious challenges [5,6,7,8,9,10]. These conditions were selected for this review because they represent distinct models of chronic immune and inflammatory dysregulation that may critically influence host responses during sepsis. These comorbidities disrupt intracellular signaling pathways, compromise endothelial integrity, and maintain chronic low-grade inflammation. The result is a preconditioned biological environment that modifies both the amplitude and regulation of the host response to subsequent infectious insults.

There is growing recognition that these baseline alterations have a pivotal role in sepsis pathogenesis [11,12,13,14,15,16,17,18]. For example, alcohol exposure disrupts gut barrier integrity, alters cytokine signaling, and impairs neutrophil function [19,20,21]. Periodontal disease amplifies the systemic inflammatory response through recurrent bacteremia and endotoxin release [22]. Psychosocial stress modifies immune cell distribution and cytokine profiles [23,24,25], while catecholamine-mediated activation of β2-adrenergic receptors (β2AR) suppresses neutrophil activity and reduces reactive oxygen species (ROS) production [26,27].

Despite considerable progress in sepsis research, the molecular and immunological mechanisms by which these comorbidities alter host responses and modulate the clinical trajectory of sepsis are not fully elucidated. This gap matters because understanding how chronic conditions reshape immune function is essential for three key reasons: accounting for the marked heterogeneity in sepsis presentation, identifying clinically relevant biomarkers, and developing more personalized therapeutic approaches. Clinical data from critically ill sepsis cohorts clearly demonstrate that comorbidities correlate with increased mortality [10], reinforcing the urgent need to dissect the underlying mechanisms.

In this review, we synthesize evidence from both experimental models and clinical studies to clarify the molecular and immunological mechanisms through which prevalent comorbidities influence sepsis progression. Our focus is placed on specific mechanisms by which these conditions modify immune responses and clinical outcomes, including mortality, immune modulation, and organ dysfunction.

Current data suggest that comorbidities do not simply coexist with sepsis; they actively reshape host immune regulation and systemic homeostasis before infection even occurs [10,11]. Many chronic conditions are characterized by persistent low-grade inflammation and sustained cellular stress, resulting in continuous release of endogenous danger signals. This environment primes the immune system, alters innate immune recognition and inflammatory signaling, perturbs endothelial and microvascular homeostasis, disrupts metabolic and mitochondrial function, and impairs neuroimmune and autonomic regulation, thereby contributing to the pathogenesis and progression of multiple-organ dysfunction in sepsis (MODS). Although these comorbidities are clinically heterogeneous, they converge on shared pathophysiological mechanisms, including persistent immune modulation and maladaptive inflammatory responses. This overlap contributes to the variability observed in sepsis severity and clinical outcomes [10]. To address this complex interplay, the following sections first outline the established pathophysiology of sepsis, subsequently detail the baseline immune and molecular features of each chronic comorbidity, and ultimately examine how these preconditioned pathways converge to drive sepsis progression and organ dysfunction.

## 2. Sepsis and MODS

In the Sepsis-3 consensus, sepsis is described as a potentially fatal condition arising from a maladaptive host response to infection, which leads to acute impairment of organ function. Clinically, organ dysfunction is defined by a rise of 2 points or more in the SOFA score, indicating failure of one or more organ systems [3]. Sepsis is now understood as a multifaceted syndrome involving simultaneous pro- and anti-inflammatory signaling, endothelial and microvascular dysfunction, metabolic shifts, coagulation disturbances, and neuroendocrine imbalance [2,3,10].

### 2.1. Innate Immune Dysregulation in Sepsis

The innate immune system constitutes the body’s first line of defense, providing rapid recognition and initial elimination of pathogens without prior antigenic exposure or immunological memory [28,29]. Key innate effector cells, including macrophages, neutrophils, dendritic cells (DCs), and natural killer (NK) cells, express pathogen-associated molecular patterns (PAMPs) to sense molecular signatures of pathogens or tissue damage [30,31]. Among pattern recognition receptors (PRRs), toll-like receptors (TLRs) are the most extensively characterized pathways linking microbial sensing to inflammatory gene activation. Notably, TLR2, TLR4, and TLR9 are most closely implicated in sepsis pathogenesis [31,32,33]. Activation of TLRs by PRRs or damage-associated molecular patterns (DAMPs) initiates intracellular signaling cascades, primarily via MyD88- and TRIF-dependent pathways, that culminate in the expression of inflammatory cytokines and chemokines [31]. Other PRRs, such as nucleotide-binding oligomerization domain (NOD)-like receptors (NLRs), contribute to innate immune detection of infection. NOD-, LRR-, and pyrin domain-containing protein 3 (NLRP3) inflammasome is not a direct receptor for microbial motifs but is activated by DAMPs, serving as a general sensor of cellular stress and damage, leading to caspase-1 activation and increased release of interleukin (IL)-1*β* and IL-18 [34,35,36,37,38,39].

Neutrophils are critical first responders to infection; inflammatory signals and PRR engagement trigger neutrophil activation. Recruitment of neutrophils to sites of infection is tightly regulated by adhesion molecules such as integrins, intercellular adhesion molecule (ICAM-1), vascular cell adhesion molecule (VCAM-1), and selectins, and by a chemokine gradient signaling through C-X-C Motif Chemokine Receptor 2 (CXCR2) receptors on circulating neutrophils that directs chemotaxis toward the site of infection [40,41,42]. These mechanisms are essential for pathogen control and tissue protection. Upon arrival, neutrophils act through phagocytosis, ROS generation, degranulation, and the release of neutrophil extracellular traps (NETs), complexes of decondensed chromatin, histones, and antimicrobial enzymes, which represent a specialized antimicrobial strategy [40,41,43].

The host response in sepsis is characterized by a dynamic interplay of pro- and anti-inflammatory processes, traditionally conceptualized as the systemic inflammatory response syndrome (SIRS) and compensatory anti-inflammatory response syndrome (CARS), which may coexist from the earliest stages [44,45]. Persistent inflammation ultimately leads to loss of systemic immunological control and organ dysfunction, accompanied by elevated cytokines (tumor necrosis factor-alpha [TNF-α], IL-1β, IL-6, IL-4, IL-12, IL-17, IL-18, IL-33) and chemokines (C-X-C Motif Chemokine Ligand-12 [CXCL12], CXCL8), as well as activation of neutrophils, complement, and coagulation pathways [46,47,48,49]. If peripheral inflammation is not contained, cytokines, chemokines, and microbial products cross the blood–brain barrier, triggering microglial and astrocyte activation, disruption of barrier integrity, increased infiltration of inflammatory cells, and enhanced ROS production [50].

Altogether, disruption of innate immune signaling pathways leads to excessive cytokine and chemokine release, neutrophil dysfunction, and a dysregulated inflammatory response, which contribute to subsequent vascular, metabolic, and organ-specific complications observed in severe sepsis.

### 2.2. Endothelial Dysfunction in Sepsis

The vascular endothelium regulates microcirculation and maintains vascular barrier integrity. Endothelial cells modulate vascular tone, blood flow, coagulation, leukocyte trafficking, and vascular permeability by releasing multiple vasoactive and immunomodulatory mediators [51]. The glycocalyx protects against excessive inflammatory and thrombotic activation [52]. During sepsis, the endothelium becomes a primary target of systemic inflammation, resulting in vascular and microcirculatory dysfunction [53].

Under pathological conditions, endothelial dysfunction is marked by a procoagulant, proadhesive, and vasoconstrictive state, accompanied by increased vascular permeability and inflammatory activation [51]. In sepsis, this dysfunction not only leads to microcirculatory impairment, glycocalyx degradation, oxidative stress, and disruption of the endothelial barrier but also accelerates progression toward MODS [53,54,55]. This process is initiated, in part, by the activation of endothelial cells by microbial components or pro-inflammatory cytokines, which is associated with activation of nuclear factor-κB (NF-κB) and NLRP3 signaling pathways. As a consequence, these cells increase the expression of inflammatory cytokines, such as TNF-α and IL-6, and of adhesion molecules, including ICAM-1, VCAM-1, and E-selectin, promoting leukocyte rolling, adhesion, and transmigration [56,57]. Concomitantly, endothelial activation induces a procoagulant phenotype characterized by increased factor expression, reduced anticoagulant activity, and impaired fibrinolysis, favoring early microvascular thrombosis [53,54]. At the same time, excessive production of ROS and reactive nitrogen species (RNS) impairs mitochondrial function and contributes to endothelial apoptosis. These changes also disrupt junctional proteins such as VE-cadherin and occludin, compromising vascular barrier integrity [58].

Glycocalyx degradation further amplifies endothelial dysfunction. Inflammatory mediators and oxidative stress promote shedding of glycocalyx components, leading to the release of syndecan-1 and heparan sulfate into the circulation. Increased circulating levels of these molecules have been associated with sepsis severity and endothelial damage [59,60]. As endothelial injury becomes sustained, persistent leukocyte infiltration together with progressive microvascular thrombosis further compromises tissue perfusion, contributing to edema formation and organ dysfunction [54,61].

Nitric oxide (NO) plays a dual role during sepsis. Excessive NO production leads to vasodilation, vascular hyporesponsiveness, and hypotension, which are key features of septic shock [62]. Additionally, NO modulates leukocyte–endothelial interactions by downregulating adhesion molecule expression, which may impair neutrophil recruitment to infection sites and contribute to systemic dissemination of infection [63]. Conversely, NO is critical for antimicrobial defense during sepsis, supporting neutrophil microbicidal activity and pathogen clearance. Experimental studies indicate that inducible nitric oxide synthase (iNOS)-deficient animals exhibit increased mortality during sepsis, while transient pharmacological modulation of iNOS activity can enhance neutrophil recruitment and local infection control [64]. Thus, dysregulated NO signaling contributes to both vascular collapse and the imbalance between antimicrobial defense and inflammatory injury observed in sepsis.

The endothelium is central to the interplay between inflammation and coagulation during sepsis. Inflammatory cytokines induce a procoagulant endothelial phenotype characterized by increased expression of tissue factor and von Willebrand factor together with suppression of endogenous anticoagulant pathways, including reduced thrombomodulin expression, impaired protein C activation, and downregulation of tissue factor pathway inhibitor (TFPI). These alterations promote thrombin generation, platelet activation, and microvascular thrombus formation. Concomitantly, increased endothelial expression of plasminogen activator inhibitor-1 (PAI-1) suppresses plasmin-mediated fibrin degradation, resulting in defective fibrinolysis. As a result, this prothrombotic and antifibrinolytic state promotes disseminated intravascular coagulation (DIC), consumption of coagulation factors, impaired tissue perfusion, and progression to MODS [65].

### 2.3. Metabolic Reprogramming and Mitochondrial Failure in Sepsis

Mitochondria play a central role in cellular bioenergetics through adenosine triphosphate (ATP) production via oxidative phosphorylation (OXPHOS). In addition to energy generation, these organelles participate in redox signaling, calcium (Ca^2+^) homeostasis, and regulation of apoptosis [66,67]. Mitochondrial integrity is essential for cellular homeostasis and for the regulation of inflammatory and antimicrobial responses [68,69]. In this context, growing evidence supports a role for mitochondrial dysfunction in the pathogenesis and progression of sepsis [70,71].

During the early stages of sepsis, tissue hypoperfusion, fluid loss, myocardial depression, and reduced vascular tone impair oxygen delivery to tissues [67]. Reduced oxygen availability, together with inflammation-induced mitochondrial dysfunction, compromises OXPHOS, resulting in decreased ATP synthesis, ionic imbalance, and excessive ROS production [67,72]. In parallel, acute systemic inflammation is associated with downregulation of genes involved in mitochondrial protein transcription and respiratory chain activity [71,73]. Together, these alterations impair mitochondrial bioenergetics and contribute to cellular dysfunction during sepsis.

Metabolic reprogramming is a hallmark of the host response in sepsis, characterized by a systemic shift from OXPHOS to aerobic glycolysis, also known as the Warburg effect [74,75]. Initially, this adaptation supports rapid immune activation and the production of inflammatory mediators. This shift involves an intentional interruption of the tricarboxylic acid cycle, leading to the accumulation of succinate, which acts as an endogenous signal to stabilize Hypoxia-inducible factor 1-α and enhance pro-inflammatory gene transcription [74,75]. However, glycolysis is less efficient for ATP generation, and a sustained reduction in OXPHOS levels results in an energetic challenge for cells in affected organs, a phenomenon described as cytopathic hypoxia [71].

As sepsis progresses, this impaired ATP production critically contributes to MODS [76]. A central mechanism for this persistent mitochondrial failure is the downregulation of the Peroxisome Proliferator-Activated Receptor Gamma Coactivator 1-alpha (PGC-1α)/Nuclear Respiratory Factor 1 (NFR1)/Mitochondrial Transcription Factor A (TFAM) axis, the key regulatory pathway of mitochondrial biogenesis [77]. The ‘TFAM paradox’ observed in sepsis indicates that while nuclear TFAM signaling may increase, a breakdown in the mitochondrial protein import machinery leads to a reduction in matrix TFAM protein, resulting in mitochondrial DNA (mtDNA) depletion and a failure to recover respiratory chain activity [77]. Alterations in fatty acid oxidation further compromise cellular energy metabolism. In addition to bioenergetic impairment, these metabolic disturbances favor the transition from an early hyperinflammatory response to a later immunosuppressive state [75].

Metabolic reprogramming is a fundamental driver of immune cell plasticity. While M1-polarized cells rely on glycolysis even under normoxic conditions, M2 macrophages depend on oxidative metabolism [78]. The importance of this mitochondrial commitment is evidenced by the loss of nicotinamide adenine dinucleotide (NADH): ubiquinone oxidoreductase subunit S4 (NDUFS4); deficiency of this Mitochondrial Complex I subunit drives macrophages toward an M1 phenotype while impairing their M2-associated repair functions [79]. This dependency extends to the adaptive arm of the immune system, where NDUFS4 proves essential for T-cell metabolic fitness. Recent evidence indicates that its absence triggers mitochondrial ROS accumulation and blunts T-cell receptor-driven activation [80]. Consequently, such persistent mitochondrial dysfunction serves as a molecular signature of sepsis, linking bioenergetic failure to immune exhaustion.

Oxidative stress is another major contributor to mitochondrial injury during sepsis. Increased production of ROS and RNS occurs through activation of nicotinamide adenine dinucleotide phosphate (NADPH) oxidases, mitochondrial dysfunction, and excessive NO generation by iNOS [81,82]. Elevated levels of these reactive species damage mitochondrial proteins, lipids, and DNA, thereby compromising respiratory chain activity and amplifying oxidative stress [82]. Mitochondrial ROS also participate in activation of the NLRP3 inflammasome, acting together with Ca^2+^flux and ionic imbalance to amplify inflammatory signaling during sepsis [83,84,85]. This may establish a self-amplifying cycle in which mitochondrial dysfunction further enhances ROS generation, progressively aggravating cellular injury and metabolic failure [86].

Sepsis also directly affects mitophagy, a specialized form of autophagy that removes damaged mitochondria. This mitophagy is coordinated by proteins such as PTEN-induced kinase 1, Parkin (an E3 ubiquitin ligase), AMP-activated protein kinase, and sirtuins, and represents an important protective mechanism for maintaining cellular energy homeostasis [75,87,88]. However, persistent inflammation and oxidative stress may impair or overload mitophagy during sepsis, resulting in the accumulation of dysfunctional mitochondria and worsening respiratory chain dysfunction [84]. Evidence suggests that mitophagy may be differentially regulated by cell type and organ. In immune cells, impaired mitophagy has been associated with sustained inflammatory activation, whereas increased mitophagic activity has been observed in other tissues during sepsis [89,90]. Although initially protective, excessive or sustained mitophagy may contribute to mitochondrial depletion, impaired ATP production, and organ dysfunction when mitochondrial biogenesis is insufficient to restore cellular energetic demands [88].

In later stages of sepsis, impairment of mitochondrial dynamics, including fission, fusion, and biogenesis, contributes to the accumulation of fragmented and dysfunctional mitochondria [75,91]. Excessive ROS production also promotes mitochondrial membrane permeabilization, facilitating the leakage of mtDNA into the cytosol and, subsequently, into the extracellular environment [92,93]. Mitochondrial injury may also promote the release of other mitochondrial DAMPs, including N-formyl peptides, which activate PRRs, such as formyl peptide receptor 1, and amplify innate immune signaling.

Recent clinical evidence demonstrates that plasma N-formyl-methionine levels are increased in septic shock, reflecting mitochondrial dysfunction and correlating with mortality [94]. In parallel, extracellular mtDNA activates inflammatory pathways such as TLR9 and the cyclic GMP-AMP synthase-stimulator of interferon genes signaling pathway, further amplifying systemic inflammation [92,95,96]. These events may culminate in apoptosis, immune dysfunction, and progression to organ failure.

The resulting mitochondrial dysfunction disrupts cellular energy homeostasis, impairs effective immune responses, and is increasingly recognized as a decisive factor in the persistent bioenergetic failure that underpins MODS in sepsis.

### 2.4. Multiple-Organ Dysfunction Syndrome

MODS refers to the acute failure of two or more organ systems, which may be reversible with timely and appropriate intervention [65]. In the intensive care setting, MODS represents the most severe manifestation of a dysregulated host response and remains a primary driver of hospital mortality [3,65]. As outlined in the Sepsis-3 consensus [3], this dysfunction is objectively quantified by the SOFA score, originally developed by Vincent et al., which assesses six key organ systems: respiratory, cardiovascular, renal, hepatic, neurological, and hematological (coagulation) [3,97].

The development of MODS in sepsis represents the convergence and amplification of the mechanisms detailed in the preceding sections, namely, ‘innate immune dysregulation in sepsis’, ‘endothelial dysfunction in sepsis’, and ‘metabolic reprogramming and mitochondrial failure in sepsis’. Through these interconnected processes, an intense inflammatory response characterized by a cytokine storm, loss of the endothelial barrier with microvascular thrombosis, and mitochondrial dysfunction collectively drive widespread and dynamic organ injury.

At the molecular level, dysregulated signaling pathways, including mitogen-activated protein kinase (MAPK) and p53, are activated during MODS and contribute to inflammatory amplification and tissue injury [98]. Experimental studies have demonstrated that pharmacological inhibition of MAPK pathways attenuates the development of MODS by reducing systemic levels of pro-inflammatory cytokines (e.g., TNF-α and IL-1β) and adhesion molecules (e.g., ICAM-1 and P-selectin) in the lung and intestine, thereby decreasing neutrophil infiltration and the rate of apoptosis and tissue damage in these organs [99]. Moreover, the stress response protein GADD45A, regulated by p53 and involved in DNA repair and cellular stress signaling, is upregulated in the kidneys and livers of mice subjected to experimental MODS and may modulate the interplay between inflammation and cell death [100]. In addition, mitochondrial dysfunction, previously discussed as a consequence of both sepsis and chronic comorbidities, plays a critical role in the progression of MODS by aggravating cellular energy deficits and amplifying organ injury [70,71,101,102,103,104].

Clinical and experimental evidence highlight the prognostic relevance of inflammatory mediators in MODS [65,105,106]. Beyond its role in early tissue injury, IL-6 emerges as a strong biomarker for predicting organ failure. Current evidence indicates that, in patients with severe trauma, the prognostic utility of IL-6 for MODS exceeds that of age, sex, or initial Injury Severity Score [65,107]. Systemic increases in IL-8 and TNF-α are recognized as defining characteristics of the acute stage of MODS [108]. In contrast, sustained elevations of interferon-γ (IFN-γ) have been shown to predict the ongoing progression toward sepsis-induced organ dysfunction [109]. Moreover, neutrophil dysfunction, characterized by impaired migration, excessive activation, and NET release, has emerged as a central driver of inflammation, microvascular thrombosis, and tissue damage, which underlie the development and progression of MODS in sepsis [41,46,110]. Taken together, these increased pro-inflammatory responses demonstrate the disruption of physiological homeostasis and exacerbate the clinical severity of MODS [65]. Organ dysfunction in MODS is typically multisystemic and dynamic:

• Pulmonary system: it is often the earliest and most prominent feature, ranging from mild hypoxemia to acute respiratory distress syndrome (ARDS). The clinical hallmark is a marked reduction in lung elasticity, resulting from the combined effects of surfactant dysfunction and alveolar fluid accumulation. These alterations compromise the physiological mechanisms necessary for effective gas exchange [111]. This pathophysiology is further exacerbated by previously described alterations in neutrophil function, such as decreased deformability and excessive NET formation [106,110]. As outlined in earlier sections, while NETs are essential for pathogen containment at sites of infection, their excessive and systemic release in sepsis leads to microvascular occlusion, sustained inflammation, and the propagation of organ injury [106,110,112].
• Cardiovascular System: The heart is the second most frequently affected organ during MODS [113,114]. While excessive NO production is a recognized contributor to septic myocardial dysfunction, the pathophysiology is multifactorial, involving innate immune activation, cytokine storm (e.g., TNF-α, IL-1β, IL-6), mitochondrial dysfunction, and catecholamine-driven β-adrenergic receptor activation [115,116]. TLRs, especially TLR4, are expressed on cardiomyocytes and endothelial cells, and their activation by PAMPs and DAMPs in sepsis triggers NF-κB signaling, cytokine release, and further myocardial depression [117,118]. Mitochondrial dysfunction, characterized by impaired oxidative phosphorylation and excessive ROS generation, also contributes to contractile failure and enhances susceptibility to cell death pathways [102,119]. The convergence of these pathways culminates in severe cardiovascular failure, marked by fluid-refractory hemodynamic instability, reduced vascular tone, endothelial barrier disruption, systemic edema, and impaired tissue oxygen delivery [111,113].
• Hepatic System: Under shock conditions, reduced hepatic perfusion rapidly induces early liver dysfunction, which may progress to sustained hepatic injury if hypoperfusion persists [111,114]. Beyond hemodynamic instability, severe sepsis causes bioenergetic collapse in the liver, marked by a significant decline in the mitochondrial Respiratory Control Ratio (RCR) and impaired ATP synthesis, driven by excessive proton leakage and disrupted Ca^2+^ homeostasis [70,106]. These mechanisms collectively contribute to hepatic failure and coagulopathy, which are characteristic of advanced organ failure [3,106].
• Renal System: In the renal system, MODS is characterized by a significant reduction in glomerular filtration rate, leading to azotemia, which acts as a systemic metabolic driver of insulin resistance and oxidative stress [3,120]. Beyond waste excretion, impairment of the kidney’s endocrine functions, including abnormal renin production and defective vitamin D activation, further disrupts ionic homeostasis and aggravates systemic metabolic instability [106,113,121]. Pro-inflammatory mediators, such as TNF-α, IL-6, and IL-1β, exacerbate this process by triggering microvascular dysfunction and tubular epithelial cell apoptosis [106].
• Neurological and Neuroendocrine Dysfunction: Sepsis-induced central nervous system dysfunction, often clinically manifested as sepsis-associated encephalopathy (SAE), results from interconnected mechanisms, including blood–brain barrier disruption mediated by matrix metalloproteinases (MMPs) and endothelial injury, microglial and astrocytic activation, neuroinflammation, and impaired cerebral microcirculation [50,122,123]. At the cellular level, mitochondrial oxidative stress, neuronal apoptosis, and neurotransmitter imbalance (cholinergic deficit and glutamate excitotoxicity) significantly impair synaptic plasticity and cognitive function. From a neuroendocrine perspective, severe MODS involves not only dysregulation of the hypothalamic–pituitary–adrenal (HPA) axis and sustained sympathetic overactivity, but also neurohypophyseal vasopressin depletion, which collectively contribute to altered cortisol dynamics, impaired autonomic regulation, vasoplegia, and hemodynamic instability [124,125,126].
• Hematological and Coagulation Systems: In addition to thrombocytopenia, MODS is frequently characterized by profound disturbances in the coagulation cascade, including increased levels of PAI-1, which suppresses fibrinolysis and promotes microvascular thrombosis, contributing to organ ischemia [61,127].

In summary, the severity and trajectory of MODS are determined by the cumulative impact of dysregulated immune responses, endothelial injury, metabolic and mitochondrial failure, and neuroendocrine disturbances. Early recognition and intervention remain critical, as MODS may be reversible with prompt supportive care, but often progresses to irreversible organ failure and death. The heterogeneity in MODS severity and clinical course reflects not only the characteristics of the infectious insult but also patient-specific factors such as comorbidities [2,3,10].

## 3. Molecular and Pathophysiological Basis of Comorbidities

### 3.1. Hypertension

Hypertension represents a complex, multifactorial disorder that significantly contributes to cardiovascular morbidity and mortality worldwide [128]. Traditionally, our understanding has centered on classical mechanisms: autonomic regulation of vascular tone [129], the renin–angiotensin–aldosterone system (RAAS) [130] and renal sodium handling [131]. However, accumulating evidence demonstrates that inflammation is a critical contributor to both the development and maintenance of hypertension. Inflammatory processes promote vascular remodeling and alter the structure and function of the heart and kidneys [132,133,134]. This chronic inflammatory state in hypertension is reflected in persistent activation of immune and vascular signaling pathways.

Several molecular and cellular pathways converge to create a pro-inflammatory milieu in hypertension. RAAS overactivation, oxidative stress, endothelial dysfunction, deregulated homocysteine metabolism, and dysregulated neuroimmune communication activate immune pathways and perpetuate inflammation, leading to vascular injury [135,136,137,138].

Angiotensin II, the principal effector of RAAS, mediates not only vasoconstriction and sodium retention but also pro-inflammatory signaling. Angiotensin II activates NADPH oxidase enzymes and increases superoxide anion (O_2_^−^) production. O_2_^−^ then reacts with NO to form peroxynitrite (ONOO^−^), which in turn uncouples endothelial nitric oxide synthase (eNOS) function. Under inflammatory conditions, iNOS becomes upregulated, producing excess NO that further promotes ONOO^−^ formation. This cascade sustains endothelial dysfunction and inflammation by maintaining activation of NF-κB, perpetuating impaired vasodilation and ongoing vascular injury [15,139,140].

Angiotensin II also promotes T-cell recruitment and activation in the vasculature, which contributes to endothelial dysfunction. Guzik et al. demonstrated that mice infused with angiotensin II show increased accumulation of CD4^+^ T lymphocytes in the adventitia and perivascular adipose tissue (PVAT). Importantly, blocking TNF-α produced by these T cells prevents both the hypertension and vascular O_2_^−^ generation induced by angiotensin II [141].

Mitochondrial dysfunction further amplifies this vascular injury. Accumulation of ROS, DNA damage, Ca^2+^overload, hypoxia, and oxidized low-density lipoprotein (LDL) disrupts the delicate balance between pro-apoptotic proteins, such as BCL-2-associated protein X (BAX), and anti-apoptotic proteins, including B-cell lymphoma 2 (BCL-2), toward apoptosis. This triggers the release of cytochrome-c and activates caspases-9 and -3, culminating in endothelial cell death [142,143,144].

The neuroimmune axis connects the sympathetic nervous system (SNS) with immune-driven vascular inflammation. Sympathetic activation stimulates monocytes, macrophages, DCs, and T lymphocytes to release ROS and pro-inflammatory cytokines, including TNF-α, IL-6, IL-1β, and IFN-γ. These mediators recruit additional immune cells, perpetuate tissue injury, and compromise vascular and renal function [145,146,147]. Peripherally produced cytokines and ROS can signal back to the central nervous system, amplifying sympathetic activity in a feed-forward loop [129,146].

In hypertension, several DAMPs are produced, including high mobility group box 1 (HMGB1), heat shock proteins, such as HSP70, extracellular ATP, uric acid crystals, and S100 proteins [15,134,148,149,150]. These molecules originate from stressed or damaged endothelial cells, vascular smooth muscle cells, macrophages, cardiomyocytes, PVAT cells, and neutrophils. PRRs expressed in immune and vascular cells recognize these DAMPs and initiate pro-inflammatory responses, resulting in the release of cytokines such as TNF-α, IL-1β, IL-6, IL-15, IL-18, IFN-γ, and IL-17, while also increasing, to a lesser extent, the expression of the anti-inflammatory cytokine IL-10 [15,149,151].

Concurrently, expression of cell adhesion molecules (CAMs), including ICAM-1, VCAM-1, and E-selectin, increases, perpetuating the cycle of vascular inflammation and dysfunction characteristic of the hypertensive state. This aligns with evidence demonstrating the role of inflammatory mediators and DAMP–PRR signaling in endothelial cells [134,148].

Hyperhomocysteinemia represents an independent risk factor for hypertension [135,152,153]. Elevated homocysteine levels enhance NADPH oxidase activity, trigger TLR-4 signaling, and activate NF-κB, driving increased expression of IL-1β, IL-6, and TNF-α in vascular cells [143,154]. Chronic exposure to inflammatory mediators promotes mitochondria-dependent apoptosis [143].

Together, these mechanisms indicate that hypertension establishes a baseline state of inflammation, mitochondrial and endothelial dysfunction, and neuroimmune remodeling.

### 3.2. Metabolic Syndrome

MetS encompasses a cluster of interconnected cardiovascular and metabolic abnormalities [155,156]. Impaired glucose regulation is one of its defining features, whereas type 2 diabetes mellitus (T2DM) is a closely associated metabolic disorder that shares key underlying pathophysiological mechanisms, including insulin resistance and chronic low-grade inflammation [155,157,158,159].

Individuals with obesity and T2DM exhibit elevated levels of circulating leukocytes, plasma coagulation factors, serum amyloid A (SAA), C-reactive protein, and pro-inflammatory cytokines such as TNF-α, IL-1β, IL-6, and chemokines. These pro-inflammatory mediators have been directly associated with insulin resistance and MetS [14,74,159,160,161,162]. Multiple studies have shown that, in obesity and T2DM, tissues such as adipose tissue, pancreas, liver, and skeletal muscle exhibit a pronounced inflammatory profile [14]. A particularly consistent observation in obesity associated with MetS or T2DM is the infiltration of macrophages into these tissues [11,14]. Under these conditions, macrophage polarization shifts from the M2 to the M1 phenotype [163,164]. Classically activated M1 macrophages produce an array of pro-inflammatory cytokines, including TNF-α, IL-6, and IL-1β [11,163]. These cytokines can induce insulin resistance by inhibiting serine phosphorylation of insulin receptor substrate proteins through IκB kinase-β and JUN N-terminal kinase pathways [11,158]. Fatty acids appear to act as triggers for classical macrophage activation in obesity, at least in part by activating TLR4 [165].

Interestingly, during the development of obesity, CD8^+^ T lymphocytes infiltrate adipose tissue before substantial macrophage accumulation occurs. These lymphocytes play a crucial role in establishing the inflammatory environment by activating resident macrophages and recruiting monocytes from the circulation, which subsequently differentiate into macrophages [166,167]. Additionally, adipose tissue from obese individuals contains elevated numbers of CD4^+^ T helper (Th) 1 lymphocytes, which secrete IFN-γ and thereby drive macrophage polarization toward the M1 phenotype. In contrast, regulatory T cells and CD4^+^ Th2 lymphocytes, which normally exert anti-inflammatory and regulatory functions to maintain adipose tissue homeostasis, are present in reduced numbers compared with other lymphocyte subsets [167,168]. Together, these immune alterations contribute to both the establishment and perpetuation of a chronic inflammatory state.

Another critical component in the pathophysiology of obesity and T2DM is the NLRP3 inflammasome. In animal models of obesity, IL-1β levels are elevated in adipose tissue, and *in vitro* studies demonstrate that NLRP3 activation in macrophages exposed to lipotoxic signals drives IL-1β production [169]. These findings are consistent with increased expression of NLRP3 inflammasome components in adipose tissue from obese individuals, thereby linking this pathway to insulin resistance and MetS [14,169].

In addition to chronic inflammation, the persistent hyperglycemia frequently present in T2DM promotes the spontaneous auto-oxidation of glucose that contributes to the formation and tissue accumulation of Advanced Glycation End products (AGEs) [170]. AGEs bind to the Receptor for Advanced Glycation End Products (RAGE), which is naturally expressed in cells such as macrophages, monocytes, DCs, and T lymphocytes. However, its expression increases significantly under chronic inflammation, such as in MetS [170].

Crucially, RAGE functions as a multiligand PRR. In addition to AGEs, it recognizes various DAMPs that are also elevated in hypertensive and stressed states, including HMGB1 and S100 proteins [149]. RAGE activation sustains inflammatory and oxidative stress responses through the NF-κB pathway, establishing a positive feedback mechanism that perpetuates the inflammatory cycle [149], driven by macrophage polarization toward the pro-inflammatory M1 phenotype, resulting in the secretion of mediators such as TNF-α, IL-6, and IL-1β [11,163,170]. Importantly, this continuous RAGE activation provides the essential ‘priming’ signal for the NLRP3 inflammasome. NLRP3, when primed, activates caspase-1 and releases high levels of active IL-1β in response to a secondary stimulus [169,171].

Chronic metabolic disorders thus represent a state of persistent immune reprogramming. Continuous exposure to metabolic stress signals, including nutrient excess, lipotoxic intermediates, and inflammasome activation, sustains innate immune activation and disrupts immune regulatory balance. Moreover, oxidative stress reduces NO availability and contributes to endothelial dysfunction in individuals with obesity [172].

In summary, these molecular alterations establish a persistent pro-inflammatory and immune-altered state in MetS and diabetes.

### 3.3. Alcohol Exposure and Alcohol Use Disorder

Alcohol use disorder (AUD) is consistently associated with impaired immune competence and an increased susceptibility to infectious diseases. Clinical studies have documented a strong association between excessive ethanol consumption and a higher incidence of bacterial and viral infections [19].

Mechanistically, however, the effects of ethanol depend on the pattern and duration of exposure. Experimental studies indicate that acute and chronic ethanol exposure are widely recognized as immunosuppressive, affecting innate and adaptive immune pathways [173,174].

Chronic alcohol consumption promotes oxidative stress, persistent inflammatory activation, and endothelial dysfunction [17,19,173,175]; ethanol generates ROS through multiple pathways, including mitochondrial dysfunction and cytochrome P450 2E1 induction, leading to lipid peroxidation, protein oxidation, and disruption of redox homeostasis [101].

These processes impair endothelial NO signaling, promote vascular inflammation, and compromise microvascular integrity, thereby reducing the organism’s capacity to maintain homeostasis [17,19,175].

Beyond its vascular effects, chronic alcohol exposure disrupts epithelial barrier integrity, particularly within the gastrointestinal tract. Increased intestinal permeability facilitates microbial translocation and sustained systemic exposure to PAMPs, including endotoxin [19,174]. This persistent low-grade endotoxemia continuously activates innate immune receptors, such as TLR4, and triggers inflammatory signaling cascades, most notably through NF-κB, contributing to systemic immune dysregulation [17,174].

Chronic ethanol exposure also affects antigen-presenting cell function. DCs are markedly affected by chronic ethanol exposure. Experimental models demonstrate reduced splenic DC numbers, impaired expression of co-stimulatory molecules such as CD40 and CD86, and altered cytokine secretion profiles, resulting in defective T-cell activation [176]. Consistent with these observations, chronic ethanol use also alters adaptive immunity by reducing circulating CD4^+^ and CD8^+^ T lymphocytes, reflecting impaired cell-mediated immune competence [174,177].

In contrast to the persistent immune alterations induced by chronic ethanol exposure, acute ethanol exposure primarily modulates the early innate immune responses in a dose- and time-dependent manner. Acute ethanol exposure alters the function of monocytes and circulating neutrophils recruited into tissues. Following acute ethanol exposure, suppression of pro-inflammatory mediator production has been consistently reported, including reductions in multiple cytokines and chemokines, such as TNF-α, IL-1β, IL-6, IL-12, macrophage inflammatory protein-1β (MIP-1β), and IFN-γ [16,174,176,178,179].

One of the most extensively characterized mechanisms following acute ethanol exposure involves disruption of lipopolysaccharide (LPS)–TLR4 signaling pathways in Kupffer cells. Experimental studies demonstrate a biphasic regulation of Kupffer cell responsiveness to LPS. Early exposure suppresses LPS-induced TNF-α production, interleukin-1 receptor-associated kinase (IRAK) activation, and NF-κB signaling, whereas delayed exposure enhances IRAK activity, amplifies NF-κB activation, and increases TNF-α release [174,180]. Ethanol has also been shown to downregulate hepatic TLR4 expression, further altering pathogen recognition and innate immune responsiveness. Together, these findings indicate that ethanol fundamentally reprograms innate immune signaling rather than simply suppressing inflammatory responses.

Collectively, acute and chronic ethanol exposure impair host defense through complementary but partially distinct mechanisms. Therefore, alcohol consumption, whether acute or chronic, can promote alterations in the immune system, oxidative stress, endothelial and barrier dysfunction, and impaired antigen presentation.

### 3.4. Psychosocial Stress

Physical or psychological stressors that disrupt homeostasis trigger coordinated neuroendocrine responses that profoundly influence immune regulation and host defense. Hans Selye first conceptualized stress as the dynamic interplay between damage and defense [121]. These adaptive responses are primarily mediated through activation of the SNS and the HPA axis, which together regulate immune function, metabolism, and cardiovascular homeostasis [124,181,182].

Activation of these systems leads to the release of catecholamines (epinephrine and norepinephrine) and glucocorticoids, both of which exert immunomodulatory effects. A large proportion of immune cells, including lymphocytes, monocytes, macrophages, and neutrophils, express receptors for these mediators, allowing stress signals to regulate immune cell trafficking and cytokine production directly [124,183,184]. Importantly, the duration of stress is a critical determinant of immune modulation. Acute, or short-term, stress typically lasts from minutes to hours, whereas chronic stress persists for several hours per day and recurs over weeks or months [185]. The impact of stress on the immune system varies considerably depending on both the type and duration of the stressor experienced.

Acute stress rapidly activates sympathetic outflow, leading to systemic catecholamine release that signals predominantly through β2-adrenergic receptor (β2AR) expressed on innate and adaptive immune cells, as well as endothelial cells [183,186,187,188,189,190,191,192]. At the cytokine level, β2-adrenergic signaling inhibits production of classical pro-inflammatory mediators, including TNF-α, IL-1β, IL-6, and IL-12, while increasing anti-inflammatory cytokines such as IL-10 [190,191]. In parallel, β2-adrenergic activation regulates endothelial–leukocyte interactions by reducing both the expression and functional activity of adhesion molecules, including ICAM-1, VCAM-1, and selectins, thereby impairing leukocyte adhesion and transendothelial migration and further limiting their recruitment to inflamed tissues [26,183,193]. These mechanisms establish a neuroimmune regulatory axis through which acute stress can transiently dampen inflammatory responses [26,124,187,194].

Chronic stress, in contrast, results in sustained neuroendocrine activation that progressively disrupts immune homeostasis. Prolonged activation of the HPA axis leads to sustained glucocorticoid exposure, which exerts potent anti-inflammatory and immunosuppressive effects through inhibition of NF-κB signaling, suppression of pro-inflammatory cytokine production, and impairment of leukocyte trafficking [195,196]. However, persistent stimulation induces glucocorticoid receptor resistance, reducing cellular responsiveness despite elevated cortisol levels. Consequently, chronic stress is frequently associated with elevated circulating levels of inflammatory mediators such as IL-6, TNF-α, and IL-1β, reflecting a shift toward low-grade systemic inflammation [197,198,199,200]. Similarly, continuous sympathetic activation contributes to immune dysregulation. While short-term β2-adrenergic signaling exerts anti-inflammatory effects, prolonged catecholamine exposure promotes receptor desensitization and internalization through G protein-coupled receptor kinase activation and β-arrestin-mediated signaling [201,202,203,204]. Chronic β2-adrenergic stimulation alters cytokine production, enhances myelopoiesis, and promotes the release of immature and pro-inflammatory myeloid cells into circulation [205]. This complex immune remodeling provides a mechanistic basis for the observed association between chronic stress and increased susceptibility to infection, as well as exacerbation of inflammatory and autoimmune diseases [198,206,207].

### 3.5. Periodontitis

Healthy periodontal tissue consists of gingival connective tissue and alveolar bone that support the tooth root and are covered by specialized oral epithelium. Periodontitis, or periodontal disease, is a chronic inflammatory condition in which bacterial plaque biofilm accumulates on tooth and root surfaces, leading to progressive destruction of periodontal connective tissue and alveolar bone, representing the leading cause of tooth loss worldwide [18,120,208,209,210].

Current understanding of periodontitis pathogenesis indicates that it results from a polymicrobial disruption of host homeostasis, driven by complex interactions between subgingival biofilms and immune responses [18,210]. While bacterial components initiate the process, the progression and amplification of tissue damage are largely driven by pro-inflammatory mediators, which orchestrate multiple downstream effects in infected periodontal tissue [211]. IL-1 plays a central role in Gram-negative bacterial infections such as periodontitis. LPS induces IL-1 production and secretion by macrophages, while TNF-α acts synergistically to amplify this inflammatory response. Additionally, IL-1 stimulates prostaglandin E2 (PGE2) synthesis in both macrophages and gingival fibroblasts by inducing cyclooxygenase-2 (COX-2) expression [212,213]. PGE2 plays a pivotal role in vasodilation and alveolar bone resorption, the latter being mediated by upregulation of the receptor activator of nuclear factor kappa-B ligand (RANKL) to osteoprotegerin (OPG) ratio. OPG is a decoy receptor that inhibits RANKL-mediated osteoclast activation [210,214,215]. Inflammatory cytokines increase RANKL expression while reducing OPG production, thereby promoting osteoclast differentiation and activity, ultimately leading to alveolar bone loss [214,216,217,218].

Furthermore, this inflammatory milieu activates vascular endothelial cells to upregulate CAM expression, most notably E-selectin and ICAM-1, facilitating leukocyte rolling, firm adhesion, and subsequent transmigration into the periodontal connective tissue [210,219,220].

The IL-23/IL-17 axis plays a pivotal role in periodontal tissue destruction. IL-23 promotes the expansion of Th17 cells, thereby increasing IL-17 production. IL-17, in turn, enhances neutrophil recruitment and upregulates the RANKL/OPG ratio, reinforcing osteoclast activation and bone resorption [221,222]. IL-17 also interacts with other inflammatory mediators such as IL-1 and TNF-α and promotes the release of matrix metalloproteinases, which degrade the extracellular matrix and exacerbate tissue destruction [223].

Collectively, these events manifest clinically as acute and chronic inflammation that promotes connective tissue destruction and alveolar bone loss, thereby increasing disease severity [223,224]. Although periodontitis is initiated by a localized microbial challenge, its consequences extend systemically. Persistent bacterial colonization and recurrent episodes of bacteremia allow microbial products, including LPS and other PAMPs, to enter systemic circulation. In parallel, locally produced inflammatory mediators such as IL-1β, TNF-α, IL-6, IL-17, and prostaglandins can disseminate systemically, contributing to sustained low-grade inflammation [22,214,218]. Chronic exposure to these circulating inflammatory signals promotes endothelial activation, increased expression of adhesion molecules, and leukocyte recruitment, contributing to systemic vascular dysfunction [225]. Moreover, repeated immune stimulation by periodontal pathogens and inflammatory mediators may induce a persistent state of immune activation and dysregulation, characterized by enhanced cytokine responsiveness and altered innate immune signaling [22].

Periodontitis thus represents not only a localized inflammatory disease but also a chronic source of systemic immune and vascular priming through continuous exposure to microbial components and inflammatory mediators [22,226,227].

**Figure 1:**
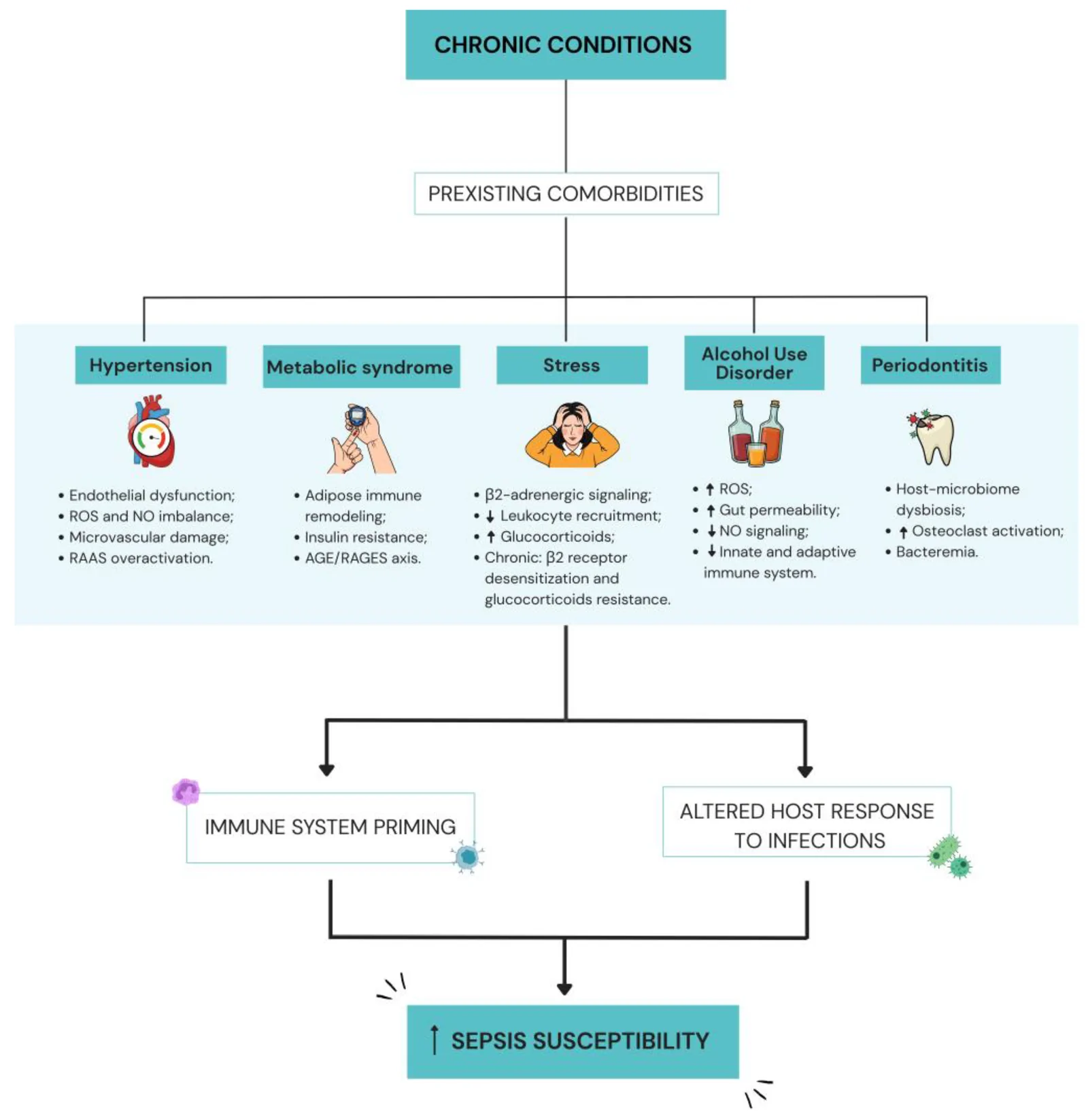
Pathophysiological mechanisms of chronic comorbidities in sepsis. This process illustrates how pre-existing conditions, such as hypertension, metabolic syndrome, stress, alcohol use disorder, and periodontitis, trigger distinct molecular and cellular changes. These baseline alterations result in endothelial dysfunction, adipose tissue remodeling, neuroimmune dysregulation, epithelial barrier disruption, and host–microbiome dysbiosis. Collectively, these pathways prime the immune system and alter the host response to infection, thereby increasing overall susceptibility to sepsis and the risk of developing multiple organ dysfunction syndrome (MODS). Created in Canva Pro (https://www.canva.com).

The interactions between comorbidities and the immune system are summarized in Figure 1.

## 4. Comorbidities and the Host Response in Sepsis

This section discusses how comorbidities modulate and amplify immune, endothelial, metabolic, and neuroimmune pathways, ultimately influencing susceptibility, severity, and clinical outcomes in sepsis and MODS [3,228]. Clinically, diabetes mellitus acts as a critical progression factor by serving as an underlying condition that alters immune function. This impairment facilitates the evolution of localized infections into systemic organ failure [2,229]. Clinical data indicate that diabetes is present in approximately 35.7% of septic patients and frequently coexists with chronic hypertension, forming profiles described as complicated multimorbidity [230,231]. Hypertension preconditions the vascular and renal systems and significantly reduces physiological reserve. This phenotype is highly associated with progression to renal failure in septic patients with multimorbidity [231]. Pre-existing vulnerability in these patients further complicates the development of sepsis-associated acute kidney injury and circulatory shock during the acute insult [228].

Chronic alcohol exposure and AUD, identified in 10–33% of critically ill patients, severely impair both pulmonary and systemic mucosal immunity [232]. AUD impairs macrophage and neutrophil function by altering chemotaxis, phagocytosis, and superoxide production. This dysfunction independently increases the risk of developing ARDS by two- to fourfold [232,233]. Clinical studies have shown that both anastomotic leakage and postoperative sepsis occur exclusively in alcohol-dependent patients, and mortality rates are markedly higher (9% vs. 0%) in those with a history of alcohol abuse, likely due to increased postoperative complications [234].

Chronic psychosocial stress also increases clinical vulnerability by disrupting neuroendocrine signaling, inducing glucocorticoid resistance, and suppressing cell-mediated bacterial clearance [235]. Clinical data demonstrate that individuals under persistent stress display delayed and significantly weaker immune responses to challenges, which places them at greater risk for severe infectious disease progression [235].

Oral chronic inflammatory foci, such as severe periodontitis, affect approximately 10% of the adult population and serve as persistent reservoirs for low-grade systemic inflammation [236]. This condition is independently associated with sustained increases in markers such as C-reactive protein and IL-6, which induce a state of trained immunity in bone marrow progenitors and lead to innate immune hyper-reactivity during subsequent inflammatory challenges, including systemic infections [236].

Taken together, these underlying conditions function as active biological drivers. Epidemiological data demonstrate that pre-existing multimorbidity can nearly double overall sepsis mortality [229,231]. In the following section, we examine the specific cellular and molecular mechanisms through which chronic preconditioning alters the host response in sepsis.

### 4.1. Innate Immune Recognition and Inflammatory Signaling

Chronic metabolic disorders, such as diabetes and obesity, induce profound baseline alterations in innate immune signaling that directly exacerbate acute septic responses. In diabetes, persistent AGE–RAGE activation and upregulation of TLR2/TLR4 create a chronically primed inflammatory state prior to infection [165,237,238]. Upon septic challenge, neutrophils from diabetic hosts exhibit heightened G protein-coupled receptor kinase (GRK2) activity, which drives the loss of surface CXCR2 expression, thereby severely impairing directed chemotaxis to the infectious focus [239,240,241,242]. Consequently, this recruitment failure leads to defective localized phagocytosis, while persistent hyperglycemia primes neutrophils for excessive NETosis, accelerating systemic tissue injury and increasing sepsis mortality [243].

Obesity and MetS similarly prime innate immunity through baseline TLR4 upregulation, M1 macrophage predominance, and NLRP3 inflammasome activation [14,165,169]. Regarding increased vulnerability to infection, leptin-deficient mice are more susceptible to pneumonia due to defective phagocytosis and bacterial killing by alveolar macrophages and neutrophils [244], despite potentially enhanced neutrophil recruitment to the lungs. Mechanistically, leptin signaling is essential for antimicrobial effector functions [245]; its absence in human and mouse neutrophils impairs the efficient assembly of the NADPH oxidase complex and reduces CD11b/CD18 cell-surface expression [244]. Consequently, this compromises ROS production, which is essential for pathogen eradication [244,246]. Collectively, a dysfunctional innate immune response in chronic metabolic conditions increases vulnerability to sepsis.

As a chronic subclinical infectious challenge, periodontal disease induces persistent activation of TLRs and cytosolic sensors (NLRP3, NOD1/NOD2), establishing a chronically primed immunological baseline [22,238]. The resulting systemic dissemination of oral pathogens and inflammatory mediators significantly lowers the host’s threshold for dysregulated hyperinflammation during subsequent septic insults [18,22].

In hypertension, chronic activation of TLR4, TLR9, and NLRP3 inflammasome pathways by endogenous DAMPs, notably HMGB1 and mitochondrial DNA, drives baseline renal and microvascular damage [15,39,247,248,249]. During secondary infectious insults, this pre-existing vascular injury synergizes with sepsis-induced NETosis to accelerate microvascular thrombosis and lower the host threshold for acute organ failure [39,112,249].

Chronic ethanol exposure impairs baseline TLR4-mediated signaling in hepatic and peripheral immune cells, predisposing the host to severe immune dysregulation during infection, as seen in murine models [174]. During infectious challenges, chronic ethanol exposure promotes excessive systemic CXCL1 levels that drive the internalization and loss of surface CXCR2 on neutrophils, thereby impairing their migration to the infectious focus [250]. In murine models of *Acinetobacter baumannii* pneumonia, chronically ethanol-exposed hosts succumb rapidly due to a failure of the neutrophil oxidative burst and of intrapulmonary TNF-α production, thereby facilitating uncontrolled bacterial replication and distal organ dissemination [250]. Paradoxically, while *in vivo* models predominantly demonstrate immune paralysis [21,250], *in vitro* data from human monocytes indicate that prolonged ethanol exposure decreases IRAK-M expression (an inhibitor of TLR signaling), thereby potentiating NF-κB signaling and cytokine release following LPS stimulation [251]. These apparently divergent findings likely reflect differences between experimental models, cell types, and the complexity of whole-organism responses versus isolated cell systems.

Psychosocial stress alters innate immune responsiveness by driving the coordinated activation of the SNS and HPA axis, predisposing the host to severe immune dysregulation during acute infections [121,124]. Acute stress increases catecholamine release that activates β2AR on innate immune cells [124], suppressing key neutrophil functions including chemotaxis, integrin expression (CD11b/CD18), and both ROS and NET generation, as shown in human neutrophils [26,27]. In experimental models, this acute stress response impairs pathogen clearance, increasing host susceptibility to infections such as *Listeria monocytogenes* [23]. Under chronic stress conditions, sustained signaling leads to β2AR desensitization and receptor internalization via GRK and β-arrestin pathways [201,203,204,252]. As adrenergic anti-inflammatory regulation is lost, combined with glucocorticoid receptor resistance induced by persistent HPA-axis activation [195], the immune system shifts toward chronic low-grade inflammation [207]. Collectively, these neuroimmune alterations establish a state of biological priming and regulatory imbalance that, in the context of a secondary septic challenge, exacerbates the dysregulated host response characteristic of severe sepsis [3,253].

In conclusion, the molecular and cellular changes induced by chronic comorbidities converge on a central theme: the disruption of innate immune homeostasis. Whether through amplified baseline inflammation (e.g., TLR and NLRP3 activation) or the loss of negative regulatory checkpoints (e.g., IRAK-M), these alterations create a permissive environment for impaired pathogen clearance and immune dysregulation, ultimately heightening the severity and unpredictability of sepsis outcomes.

### 4.2. Endothelial and Microvascular Dysfunction in Comorbid Hosts

As discussed in Section 3, chronic comorbidities create a systemic vascular environment characterized by persistent endothelial stress and impaired redox homeostasis, which compromise microvascular adaptation and prime patients for severe organ dysfunction and increased mortality during sepsis.

In hypertension, persistent RAAS activation and oxidative stress reduce NO bioavailability and promote vascular injury [138]. Diabetes and obesity contribute through chronic metabolic inflammation and activation of the AGEs/RAGE and NLRP3 pathways, which impair endothelial homeostasis and microvascular regulation [172]. Chronic alcohol exposure further aggravates endothelial impairment by increasing gut permeability, endotoxemia, and ROS production [19,101]. Meanwhile, periodontal disease and chronic psychosocial stress sustain endothelial activation through persistent inflammatory and neuroimmune alterations [22,254]. Collectively, these conditions create a chronically activated vascular environment that intensifies endothelial injury during sepsis, establishing a direct link between preexisting comorbidities and acute vascular dysfunction.

Clinical and experimental evidence supports the association between chronic endothelial dysfunction and increased susceptibility to sepsis [255]. Elevated baseline levels of endothelial activation markers, including IL-6, E-selectin, and ICAM-1, correlate with increased sepsis risk, supporting the role of endothelial dysfunction as a pathophysiological bridge between preexisting vascular disease and infection vulnerability [256]. Correspondingly, patients with diabetes and cardiovascular disease frequently present elevated circulating markers of endothelial activation [257]. These observations confirm that vascular injury is often established prior to infection onset, potentially influencing sepsis progression and clinical outcomes.

Persistent oxidative stress associated with chronic comorbidities impairs eNOS activity, thereby reducing NO bioavailability and increasing O_2_^−^generation [258]. Superoxide rapidly reacts with NO to form ONOO^−^, a highly reactive molecule that drives oxidative injury, mitochondrial dysfunction, and tissue hypoxia during sepsis. Consequently, these downstream events aggravate endothelial dysfunction and severely compromise microvascular perfusion [259,260].

Taken together, these lines of evidence indicate that chronic comorbidities do not simply represent passive risk factors; rather, they actively prime the endothelium and microvasculature for pronounced dysfunction during sepsis, thereby increasing disease severity and worsening clinical outcomes.

### 4.3. Metabolic and Mitochondrial Injury in Comorbid Hosts

Chronic comorbidities create a metabolic environment characterized by persistent mitochondrial stress and impaired redox homeostasis, which compromise cellular metabolic adaptation and predispose patients to acute organ dysfunction and increased mortality during sepsis [102,261,262].

In T2DM, persistent hyperglycemia, insulin resistance, and chronic low-grade inflammation lead to sustained metabolic imbalance and directly impact immune and mitochondrial function [102,263,264]. Diabetes is associated with increased formation of AGEs, activation of protein kinase C pathways, enhanced polyol and hexosamine flux, and excessive O_2_^−^ production, all of which contribute to oxidative stress and mitochondrial dysfunction [264,265,266]. These alterations establish a baseline immunometabolic dysfunction that impairs host immune responses and may favor infection dissemination, contributing to increased morbidity and mortality observed in diabetic patients during severe infections [267].

Obesity, insulin resistance, and T2DM are also associated with elevated circulating free fatty acids (FFAs) [268,269], which activate TLR2 and TLR4 signaling, triggering inflammatory cascades that overlap with pathways activated during sepsis [102]. Hyperglycemia and lipid overload increase ROS production via mitochondrial dysfunction, NADPH oxidase activation, and uncoupling of NO synthesis [270,271], while obesity-related chronic inflammation sustains persistent oxidative stress and mitochondrial damage [119]. Additionally, hypertension aggravates mitochondrial dysfunction through sustained RAAS activation and angiotensin II-induced ROS production, impairing redox balance and bioenergetic efficiency [272]. During sepsis, this persistent oxidative stress may further compromise bioenergetic efficiency and cellular metabolic adaptation.

Furthermore, chronic alcohol exposure triggers the release of endogenous DAMPs, such as mitochondrial ATP and uric acid, which prime the NLRP3 inflammasome–caspase-1 axis and induce mitochondrial dysfunction, persistent oxidative stress, and impaired antioxidant defenses, thereby amplifying inflammation and organ injury [19,101,273]. Chronic psychosocial stress also contributes to metabolic dysregulation, mitochondrial ROS production, and altered neuroimmune signaling, exacerbating immunometabolic dysfunction during sepsis [103]. Similarly, periodontal disease indirectly drives mitochondrial injury through persistent systemic inflammation and recurrent exposure to bacterial endotoxins [104]. Together, these conditions reinforce the concept that preexisting metabolic and inflammatory alterations actively amplify mitochondrial collapse during sepsis.

### 4.4. Neuroimmune and Autonomic Dysfunction in Comorbid Hosts

Chronic comorbidities profoundly disrupt neuroimmune and autonomic regulatory mechanisms. Persistent changes in autonomic tone, neuroimmune signaling, and gut microbial ecology are well documented in conditions such as obesity, diabetes, hypertension, chronic stress, AUD, and periodontitis [215,274,275,276,277,278,279]. Bidirectional communication between the nervous and immune systems is mediated by the autonomic nervous system, comprising both sympathetic and parasympathetic divisions, and the HPA axis [125]. The parasympathetic system, primarily via the vagus nerve, exerts anti-inflammatory effects through the “inflammatory reflex” [280]. Inflammatory mediators such as TNF-*α* and IL-1β, released during infection or tissue injury, activate afferent vagal fibers, transmitting signals to the brainstem to initiate an efferent response. This response suppresses peripheral cytokine production via the cholinergic anti-inflammatory pathway (CAP), in which acetylcholine acts on α7 nicotinic acetylcholine receptors (α7nAChR) on immune cells [280,281]. Concurrently, infection or tissue injury triggers activation of the SNS, increasing catecholamine release [192]. Norepinephrine and epinephrine bind to β2AR on a range of immune cells, including CD4^+^ T lymphocytes, monocytes/macrophages, and neutrophils [26,187]. In CD4^+^ T cells, β2AR stimulation promotes acetylcholine release, which acts on α7nAChR on macrophages to inhibit pro-inflammatory cytokine secretion and amplify the CAP [282]. In monocytes and macrophages, β2AR activation inhibits cytokine and chemokine production [283], while in neutrophils, it reduces recruitment, activation, and ROS production [26]. However, whereas acute sympathetic activation can transiently suppress inflammation, chronic sympathetic overactivity—a hallmark of obesity, hypertension, diabetes, and prolonged stress—leads to progressive β2AR desensitization and a shift toward pro-inflammatory signaling [27,124,200,201,202,203,204,252,284,285]. When sepsis develops in hosts with chronic comorbidities, preexisting adrenergic receptor desensitization and HPA-axis dysregulation, such as altered circadian cortisol rhythms and glucocorticoid receptor resistance, severely impair immunovascular control, favoring uncontrolled inflammation and organ dysfunction [126,285,286,287,288].

Recent evidence highlights the gut–brain axis as a pivotal interface in neuroimmune homeostasis [277]. Chronic inflammatory and metabolic comorbidities are frequently accompanied by intestinal dysbiosis, characterized by an increased abundance of pro-inflammatory bacterial genera (e.g., *Enterobacteriaceae*, *Fusobacterium*) and reduced levels of beneficial commensals (e.g., *Faecalibacterium*, *Bifidobacterium*) [277,289]. In obesity and diabetes, this shift enhances intestinal permeability, driving chronic low-grade elevation of circulating LPS (metabolic endotoxemia) that perpetuates systemic inflammation [290,291]. Chronic alcohol consumption induces a distinct dysbiosis, characterized by reductions in *Lactobacillus* and *Bifidobacterium* and increases in *Proteobacteria* and *Streptococcus*, further compromising gut barrier integrity and favoring the translocation of LPS and peptidoglycan [21,275,292]. Similarly, periodontitis-associated oral pathogens, such as *Porphyromonas gingivalis* and *Fusobacterium nucleatum*, alter the oral–gut–brain axis, amplifying systemic and neuroinflammatory responses [293,294].

Dysbiosis alters the microbial metabolic profile, reducing short-chain fatty acids (SCFAs; notably butyrate and propionate) and elevating trimethylamine N-oxide (TMAO) [277,294]. Decreased SCFA levels impair vagal tone and drive microglial activation and neuroinflammation, whereas elevated TMAO and LPS drive systemic and central inflammatory cascades [277,295,296,297]. This pro-inflammatory baseline primes the host for exaggerated or ineffective immune responses during secondary acute infection, increasing susceptibility to severe sepsis [298].

Taken together, chronic comorbidities exert convergent, detrimental effects on neuroimmune and autonomic regulatory networks through sympathetic hyperactivation, CAP impairment, and gut–brain axis disruption [215,274,275,276,277,278,279,280]. Consequently, these preexisting alterations undermine autonomic reflex control and prime the host for SAE, organ dysfunction, and increased mortality in sepsis [86,122,288].

### 4.5. Progression to MODS in Comorbid Hosts

As discussed in the preceding sections, the pathogenesis of MODS in sepsis is the culmination of multiple convergent mechanisms, many of which are preconditioned or exacerbated by chronic comorbidities [13,17,102,111,120,299]. Preexisting conditions such as hypertension, diabetes, obesity, chronic alcohol exposure, periodontitis, and chronic stress sustain low-grade inflammation, endothelial and microvascular injury, and cellular metabolic strain [14,15,19,200,250]. During acute sepsis, these baseline alterations lower the physiological threshold for acute tissue damage, increasing patient vulnerability to severe, less reversible manifestations of MODS [3,111].

• Pulmonary dysfunction. Sepsis-induced lung injury is aggravated by preexisting comorbidities. Hypertension and diabetes contribute to endothelial dysfunction and microvascular injury [14,15]; obesity promotes chronic low-grade inflammation and impairs respiratory mechanics [13,172]; and chronic alcohol consumption disrupts epithelial barrier integrity and neutrophil antimicrobial function [17,19,250]. Collectively, these alterations enhance susceptibility to acute lung injury, impair gas exchange, and increase the severity of respiratory failure during MODS.
• Cardiovascular dysfunction. Chronic comorbidities further destabilize cardiac function during MODS. Hypertension and diabetes promote myocardial inflammation through increased infiltration of pro-inflammatory immune cells (such as macrophages and T lymphocytes), upregulation of cytokines, including TNF-α and IL-6, enhanced oxidative stress, and activation of TLR signaling pathways, all of which contribute to cardiac dysfunction [14,15]. MetS enhances oxidative stress and impairs mitochondrial energetics [172,218]. Chronic stress and sustained catecholamine exposure promote β-adrenergic receptor desensitization via GRK2 and β-arrestin pathways, reducing cardiac responsiveness and contractile reserve [27,207,300]. There is evidence that NO excess may also induce GRK2 expression, further aggravating β-adrenergic receptor dysfunction [300]. Notably, the upregulation of GRK2 in cardiomyocytes parallels the mechanism previously discussed in this review in septic neutrophils, where NO excess impairs CXCR2-mediated trafficking [240,300,301]. This highlights GRK2 as a shared molecular mediator contributing to organ dysfunction in sepsis. The convergence of these pathways culminates in severe cardiovascular failure, defined by hemodynamic instability that fails to respond to fluid resuscitation, often accompanied by disturbances in cardiac rhythm. This clinical state is exacerbated by reduced vascular tone and disruption of endothelial barriers, which drive systemic edema and reduced oxygen diffusion to the tissues [111,114].
• Central nervous system and neuroendocrine dysfunction. Preexisting comorbidities further compromise neural homeostasis and resilience during critical illness [3,302]. Conditions such as diabetes, hypertension, obesity, AUD, and psychosocial stress, which are associated with baseline neuroimmune and autonomic dysregulation, impair blood–brain barrier integrity and promote microglial priming [3,15,123,200,277,288,295,296,297]. Furthermore, chronic sympathetic overactivity associated with obesity, hypertension, diabetes, and prolonged stress promotes β2-adrenergic receptor desensitization, whereas persistent HPA-axis activation, particularly during chronic psychosocial stress, may induce glucocorticoid receptor resistance, blunting central autonomic control and compromising cholinergic anti-inflammatory regulation [125,180,194,280]. When exposed to acute septic insults, this preconditioned neuroendocrine environment accelerates central neuroinflammation, may contribute to relative vasopressin deficiency or an impaired vasopressinergic response, and disrupts HPA-axis dynamics, predisposing patients to SAE, vasoplegia and hemodynamic instability, and accelerated progression of MODS [279,287,288,303].
• Hepatic dysfunction. Chronic comorbidities substantially lower the threshold for hepatic dysfunction during MODS. In diabetes, persistent hyperglycemia and insulin resistance promote hepatic inflammation and oxidative stress through SAA-mediated NF-κB activation [14,304]. Similarly, chronic alcohol consumption induces baseline oxidative stress and effectively arrests the liver’s regenerative process, preventing hepatocytes from progressing from G1 (prereplicative) to S (replicative) phases necessary for recovery [16,305]. Collectively, these factors exacerbate systemic inflammation and metabolic exhaustion, leading to severe hepatic failure and associated coagulopathy characteristic of MODS [3,106].
• Renal dysfunction. The presence of chronic comorbidities markedly increases renal vulnerability during MODS. Diabetes promotes chronic microvascular damage and low-grade inflammation through SAA-mediated TLR4 signaling, increasing the risk of acute kidney injury during sepsis [14,304]. Simultaneously, obesity and MetS amplify this susceptibility by disrupting adipokine balance and promoting abnormal lipid accumulation within the renal parenchyma. These alterations, together with increased systemic oxidative stress, progressively diminish the kidney’s physiological reserve against septic insults [13,14,119]. Chronic inflammation associated with hypertension induces vascular remodeling and endothelial dysfunction, which reduces the kidney’s ability to maintain stable perfusion under septic stress [15,111,134]. Chronic alcohol consumption impairs host immunity and predisposes to renal failure by promoting accelerated bacterial dissemination to the kidneys and inducing nephromegaly and glomerular swelling [250]. Notably, periodontal disease is a sustained source of systemic inflammation, creating a detrimental reciprocal relationship with the kidneys [120]. This interplay is further influenced by shared genetic predispositions, particularly functional polymorphisms in the IL-1β (IL1B) gene and its receptor antagonist (IL-1RN), which link periodontitis to an elevated risk of both chronic and acute renal dysfunction [120,306]. Finally, chronic psychosocial stress, through sustained activation of the sympathetic nervous system and the HPA axis, alters renal hemodynamics and impairs immune regulation, further predisposing the host to sepsis-associated renal failure [13,121,200]. These comorbidity-driven alterations in immune, vascular, and metabolic pathways not only increase the incidence and severity of renal dysfunction during sepsis and MODS but are also associated with a greater need for renal replacement therapy and poorer patient outcomes [111].
• Gastrointestinal dysfunction. Gastrointestinal manifestations of MODS frequently involve stress-induced mucosal injury, adynamic ileus, and feeding intolerance, which are driven by systemic inflammatory mediators and acute epithelial cell apoptosis [106,299]. These alterations are further aggravated by profound imbalances in autonomic regulation [200,307]. In particular, diminished vagal activity compromises the CAP, eliminating an essential control on cytokine synthesis, whereas concurrent sympathetic hyperactivity induces pronounced mesenteric vasoconstriction and suppresses intestinal motility [111,121,281]. These neuroautonomic disturbances directly contribute to mucosal ischemia and intestinal paralysis, processes that are especially evident in individuals with chronic comorbidities [13,16]. In diabetes and obesity, chronic hyperglycemia and low-grade inflammation further compromise the integrity of the gut barrier [14,106]. Chronic alcohol consumption imposes an additional burden by activating tyrosine kinase pathways that compromise tight junction integrity, markedly increasing paracellular permeability and promoting colonic dysbiosis [174,308]. The resulting barrier dysfunction facilitates the systemic translocation of pathobionts and bacterial products, thereby amplifying inflammation in remote organs [111,114]. This process is further linked to periodontal disease, as oral bacteria such as *Klebsiella aerogenes* can translocate to the gut, exacerbating intestinal inflammation and contributing to the progression of multi-organ failure [120,309].
• Hematologic dysfunction and coagulopathy. Prothrombotic alterations and microvascular thrombosis are particularly pronounced in patients with hypertension, diabetes, obesity, and MetS, where chronic inflammation and endothelial activation signaling via protease-activated receptors (PARs) sustain a baseline hypercoagulable state [310,311]. PAI-1 expression is upregulated by inflammatory cytokines, such as IL-6, and is closely linked to insulin resistance and central adiposity, thereby increasing the risk of disseminated intravascular coagulation in individuals with comorbidities [127]. Furthermore, metabolic diseases commonly impair the protective functions of the nuclear receptor peroxisome proliferator-activated receptor gamma (PPAR-γ). Reduced activity of this receptor diminishes its ability to suppress the expression of genes that drive inflammation and thrombosis, thereby compromising vascular stability [312]. Chronic alcohol use can further exacerbate coagulopathy by impairing hepatic production of coagulation factors and altering platelet function [19]. The combined effect of these mechanisms leads to a heightened risk of both thrombosis and bleeding complications during MODS in patients with underlying comorbidities.

Collectively, chronic comorbidities amplify the mechanisms underlying MODS by sustaining immune dysregulation, endothelial dysfunction, metabolic and mitochondrial impairment, and neuroendocrine imbalance. Persistent alterations driven by diabetes, obesity, hypertension, alcohol use, periodontitis, and chronic stress increase the severity of organ dysfunction and contribute to poorer clinical outcomes [13,15,17,102,111,299]. Understanding the mechanistic links between specific comorbidity profiles and organ dysfunction is essential for risk stratification and the development of targeted therapies [65,111]. Ultimately, these comorbidity-driven changes heighten the risk of life-threatening organ dysfunction and contribute to poorer patient outcomes and high in-hospital mortality [2,3,12].

### 4.6. Additional Comorbidities Beyond the Scope of the Present Review

The comorbidities discussed in this review were intentionally selected because they share common mechanistic pathways involving chronic inflammation, endothelial dysfunction, innate immune activation, and metabolic dysregulation. Nevertheless, other clinically relevant conditions may also influence host susceptibility to sepsis through related mechanisms, including cancer, aging, and neurodegenerative diseases.

Cancer is frequently associated with systemic immune dysregulation and an immunosuppressive microenvironment. This is partly driven by the expansion of myeloid-derived suppressor cells (MDSCs), which compromise antimicrobial immune responses and increase susceptibility to severe infections [313]. Similarly, neurodegenerative disorders, including Alzheimer’s disease and Parkinson’s disease, are characterized by persistent neuroinflammation and alterations in peripheral immune function, which together may influence systemic inflammatory responses [314]. Although aging is not a disease per se, the processes of immunosenescence and inflammaging profoundly impair both innate and adaptive immunity and promote chronic low-grade inflammation, thereby increasing vulnerability to sepsis and adverse clinical outcomes [315].

These examples further support the concept that persistent immune dysregulation and chronic inflammation, regardless of the underlying disease, may shape the host response to sepsis. Whether these conditions converge on the same mechanistic pathways discussed in this review or involve distinct biological processes remains to be determined. Addressing these questions may further refine risk stratification and the development of precision therapeutic strategies for sepsis.

## 5. Translational Framework for Risk Stratification and Precision Therapeutics

To actively operationalize this understanding, we propose an integrative translational framework that aligns pre-existing chronic conditions with the recently proposed 5 Domains of Risk model, establishing a hypothesis-generating blueprint to transition sepsis management from reactive scoring toward mechanistically informed precision care [228]. We hypothesize that chronic diseases are not passive, disconnected background variables. Instead, they impose a persistent biological overload and baseline immune reprogramming across five core pathobiological axes: immunity/inflammation, the endothelium, epithelial barriers/microbiota, the neuroaxis, and immunometabolism [3,228].

From a risk-stratification perspective, this framework can be implemented as a sequential, multi-layered prognostic model that complements traditional acute clinical scores such as SOFA, qSOFA, and SIRS [3,194,195]. First, a patient’s unique chronic comorbidity profile is mapped to identify baseline cellular and microvascular susceptibilities before infectious onset [10]. For example, chronic arterial hypertension drives a sustained pro-inflammatory milieu and endothelial priming, characterized by elevated baseline systemic levels of endothelial activation markers, including IL-6, E-selectin, and ICAM-1 [256]. Concurrently, metabolic disorders such as obesity and type 2 diabetes mellitus induce continuous RAGE signaling and low-grade tissue stress, providing the necessary biological priming signal for chronic NLRP3 inflammasome autoactivation [14,165,169,170]. By dynamically integrating these pre-existing biological signatures with hyperacute clinical parameters and organ-specific damage biomarkers (such as neutrophil gelatinase-associated lipocalin [NGAL] or kidney injury molecule-1 [KIM-1] for early renal injury, or B-type natriuretic peptide [BNP/N-terminal pro-B-type natriuretic peptide [NT-proBNP] for myocardial strain) through emerging transcriptomics, multi-omics analyses, and computational machine learning tools, clinicians could stratify highly heterogeneous patient populations into distinct, biologically informed subgroups [2,3,165,189,316]. This approach could allow prediction of specific organ vulnerabilities, such as cardiorenal flow-function dissociation or severe capillary leak syndromes, long before macroscopic organ failure manifests clinically at the bedside [184,228,317].

From a therapeutic development perspective, delineating these comorbidity-specific pathways provides a rational framework to guide host-directed interventions and overcome the limitations of uniform, rigid protocols [10,48,318]. Pre-existing pathobiological profiles can be utilized to enrich patient populations, refine inclusion criteria, and optimize pharmacokinetic and pharmacodynamic dosing regimens in future adaptive clinical trials [228,304,319]. For instance, patients presenting with a predominant endothelial or hyperinflammatory comorbidity overload could be selectively allocated to mechanism-matched therapeutic bundles, a translational strategy strongly supported by the clinical precedent of the ImmunoSep randomized trial, which demonstrated that precision immunotherapies tailored to a patient’s individual biological profile significantly reduced acute organ dysfunction [303]. Crucially, this comorbidity-tailored paradigm must be treated as a conceptual model; the clinical standardization of baseline biomarkers, the logistical challenges of deploying computational real-time analytics within narrow therapeutic windows, and the evaluation of long-term homeostatic restoration among survivors remain major hurdles that require rigorous, prospective validation in future clinical trials [3,10,228]. Ultimately, these comorbidity-driven changes heighten the risk of life-threatening organ dysfunction and contribute to poorer patient outcomes and high in-hospital mortality [2,3,10,12].

## 6. Conclusions

The evidence synthesized in this review demonstrates that chronic comorbidities, including hypertension, diabetes, obesity, psychosocial stress, AUD, and periodontitis, do not merely coexist with sepsis but actively reprogram the host environment before the onset of acute infection and can determine its pathogenesis and clinical course [3,14,15,17,309]. Through persistent alterations in innate immune signaling, endothelial and microvascular function, metabolic and mitochondrial homeostasis, and neuroimmune regulation, these conditions establish a baseline state of biological priming [3,13,15,19,206,261]. Although clinically heterogeneous, these comorbidities converge on shared pathobiological axes, promoting the persistent activation of the TLR–NF-κB and NLRP3 inflammasome pathways, thereby compromising the host’s ability to respond effectively to infectious insults and increasing both the susceptibility to and severity of sepsis [14,15,102,134,169,174,197,200,228].

In recent years, the paradigm of uniform sepsis management has been increasingly challenged [10,48]. Advances in precision medicine are based on identifying patient subgroups through clinical subphenotypes (from physiological and laboratory data) and molecular endotypes (defined by biological signatures) [2,3,106,303]. As an essential integrative tool for decoding this systemic vulnerability in multimorbidity scenarios, the 5 Domains of Risk Model [228] has emerged as a promising conceptual framework explaining how chronic diseases distinctly overload five central pathobiological axes: immunity/inflammation, endothelium, barriers/microbiota, neuroaxis, and immunometabolism. This approach enables the interpretation of clinical heterogeneity at the bedside, guiding personalized therapeutic interventions according to the patient’s individual risk phenotype [106,243]. Validating this paradigm shift, the ImmunoSep clinical trial provided initial evidence that precision immunotherapy strategies, tailored to the patient’s predominant biological profile, resulted in a significant reduction in acute organ dysfunction [303]. Therefore, sepsis management is moving beyond universal therapeutic approaches by incorporating emerging biomarkers, transcriptomics, and machine learning tools, alongside advanced translational platforms such as organ-on-chip systems [2,3]. The future of sepsis management will require a commitment that goes beyond conventional intensive care unit protocols, encompassing longitudinal monitoring, immunological rehabilitation, and the long-term restoration of host homeostasis [3,10,12].

## Data Availability

No new data were created or analyzed in this study. Data sharing is not applicable to this article.

## Abbreviations

The following abbreviations are used in this manuscript:

AGEs	Advanced Glycation End Products
α7nAChR	Alpha-7 Nicotinic Acetylcholine Receptor
ARDS	Acute Respiratory Distress Syndrome
ATP	Adenosine Triphosphate
AUD	Alcohol Use Disorder
BAX	BCL-2-associated X Protein
BCL-2	B-cell Lymphoma 2
BNP	B-type Natriuretic Peptide
β2AR	β2-Adrenergic Receptors
Ca^2+^	Calcium
CAMs	Cell Adhesion Molecules
CAP	Cholinergic Anti-inflammatory Pathway
CARS	Compensatory Anti-inflammatory Response Syndrome
COX-2	Cyclooxygenase-2
CXCL	C-X-C Motif Chemokine Ligand
CXCR2	C-X-C Motif Chemokine Receptor 2
DAMPs	Damage-Associated Molecular Patterns
DCs	Dendritic Cells
DIC	Disseminated Intravascular Coagulation
DNA	Deoxyribonucleic Acid
eNOS	Endothelial Nitric Oxide Synthase
FFA	Free Fatty Acid
GRK2	G-protein-coupled Receptor Kinase 2
HMGB1	High Mobility Group Box 1
HPA	Hypothalamic–Pituitary–Adrenal Axis
ICAM-1	Intercellular Adhesion Molecule-1
IFN-γ	Interferon-gamma
IL	Interleukin
IL-1RN	Interleukin-1 Receptor Antagonist
iNOS	Inducible Nitric Oxide Synthase
IRAK	Interleukin-1 Receptor-Associated Kinase
KIM-1	Kidney Injury Molecule-1
LDL	Low-Density Lipoprotein
LPS	Lipopolysaccharide
MAPK	Mitogen-Activated Protein Kinase
MDSCs	Myeloid-Derived Suppressor Cells
MetS	Metabolic Syndrome
MIP-1β	Macrophage Inflammatory Protein-1β
MMPs	Matrix Metalloproteinases
MODS	Multiple Organ Dysfunction Syndrome
mtDNA	Mitochondrial DNA
NADH	Nicotinamide Adenine Dinucleotide
NADPH	Nicotinamide Adenine Dinucleotide Phosphate
NDUFS4	NADH:Ubiquinone Oxidoreductase Subunit S4
NETs	Neutrophil Extracellular Traps
NF-κB	Nuclear Factor Kappa B
NGAL	Neutrophil Gelatinase-Associated Lipocalin
NK	Natural Killer
NLR	NOD-like Receptor
NLRP3	NOD-like Receptor Pyrin Domain-containing 3
NO	Nitric Oxide
NOD	Nucleotide-binding Oligomerization Domain
NRF-1	Nuclear Respiratory Factor 1
NT-proBNP	N-terminal pro-B-type Natriuretic Peptide
O_2_^−^	Superoxide Anion
ONOO^−^	Peroxynitrite
OPG	Osteoprotegerin
OXPHOS	Oxidative Phosphorylation
PAI-1	Plasminogen Activator Inhibitor-1
PAMPs	Pathogen-Associated Molecular Patterns
PARS	Protease-Activated Receptors
PGC-1α	Proliferator-Activated Receptor Gamma Coactivator 1-α
PGE2	Prostaglandin E2
PPAR-γ	Peroxisome Proliferator-Activated Receptor Gamma
PRRs	Pattern Recognition Receptors
PVAT	Perivascular Adipose Tissue
RAAS	Renin–Angiotensin–Aldosterone System
RAGE	Receptor for Advanced Glycation End Products
RANKL	Receptor Activator of Nuclear Factor Kappa-B Ligand
RCR	Respiratory Control Ratio
RNS	Reactive Nitrogen Species
ROS	Reactive Oxygen Species
SAA	Serum Amyloid A
SAE	Sepsis-Associated Encephalopathy
SCFAs	Short-Chain Fatty Acids
SIRS	Systemic Inflammatory Response Syndrome
SNS	Sympathetic Nervous System
T2DM	Type 2 Diabetes Mellitus
TFAM	Mitochondrial Transcription Factor A
Th	T Helper Cells
TLR	Toll-like Receptor
TMAO	Trimethylamine N-oxide
TNF-α	Tumor Necrosis Factor-alpha
VCAM-1	Vascular Cell Adhesion Molecule-1
